# Dynamic Tracking of Injected Mesenchymal Stem Cells after Myocardial Infarction in Rats: A Serial 7T MRI Study

**DOI:** 10.1155/2016/4656539

**Published:** 2016-08-30

**Authors:** Xiuyu Chen, Minjie Lu, Ning Ma, Gang Yin, Chen Cui, Shihua Zhao

**Affiliations:** ^1^Department of Radiology, State Key Laboratory of Cardiovascular Disease, Fuwai Hospital, National Center for Cardiovascular Diseases, Chinese Academy of Medical Sciences and Peking Union Medical College, Beijing 100037, China; ^2^Department of Ultrasound, Anzhen Hospital, Beijing 100029, China

## Abstract

*Purpose.* To track the fate of micron-sized particles of iron oxide (MPIO) labeled mesenchymal stem cells (MSCs) in vivo in a rat myocardial infarction model using 7T magnetic resonance imaging (MRI) scanner.* Materials and Methods.* Male MSCs (2 × 10^6^/50 *μ*L) dual-labeled with MPIO and CM-DiI were injected into the infarct periphery 7 days after myocardial infarction (MI). The control group received cell-free media injection. The temporal stem cell location, signal intensity, and cardiac function were dynamically assessed using a 7T MRI at 24 h before transplantation (baseline), 3 days, 2 weeks, and 4 weeks after transplantation, respectively.* Results.* MR hypointensities caused by MPIOs were observed on T2^⁎^-weighted images at all time points after MSCs injection. Cine-MRI showed that MSCs moderated progressive left ventricular remodeling. Double staining for iron and CD68 revealed that most of the iron-positive cells were CD68-positive macrophages. Real-time PCR for rat SRY gene showed the number of survival MSCs considerably decreased after transplantation. MSC-treated hearts had significantly increased capillary density in peri-infarct region and lower cardiomyocytes apoptosis and fibrosis formation.* Conclusions.* Iron particles are not a reliable marker for in vivo tracking the long-term fate of MSCs engraftment. Despite of poor cell retention, MSCs moderate left ventricular remodeling after MI.

## 1. Introduction

Stem cell-based therapy has been currently introduced as a potentially promising approach to treat myocardial infarction (MI) and heart failure. As multipotent progenitor cells, bone-marrow-derived mesenchymal stem cells (MSCs) are easy to obtain and expand in vitro and have been considered to be an ideal option for clinical and basic applications [1]. According to previous studies, MSCs transplantation was proved to be able to attenuate left ventricular remodeling and cardiac dysfunction without significant safety concerns [2, 3]. However, noticeable concerns have been raised and yet to be studied, such as the fate of transplanted cells and the underlying mechanisms of the functional benefits.

Magnetic resonance imaging (MRI) is currently considered as a standard tool to evaluate the cardiac morphology and function due to its versatility, accuracy, and reproducibility and has been most frequently applied in the clinical trials [4–8]. However, echocardiography was more commonly used instead of MRI in rodent hearts studies because of their quite small size and high heart rate (>300 beats/min) [9–13]. The measurements by echocardiography are less accurate than cine-MRI especially when applied to asymmetric, infarcted rat hearts [14–16]. Furthermore, T2^⁎^-weighted imaging is able to determine whether iron-labeled cells were successfully transplanted into the target sites of infarcted hearts as well as the initial cell retention, which is crucial for the following therapeutic effects on cardiac function and therefore makes MRI an potentially ideal monitoring imaging modality for tracking the fate of engrafted MSCs.

Our preliminary study has revealed that MRI scanner with ultra-high magnetic field (7.0T) could accurately evaluate the cardiac function and morphology in MI models of rats whereas superparamagnetic iron oxide nanoparticles were unable to reliably track the fate of engrafted MSCs [17]. However, the effect of MSCs after injection and the value of tracking MSCs by MRI remain as a controversial issue to be resolved. Hence, we aimed to use a 7.0T MRI scanner to dynamically track the fate of fluorescent micron-sized particles of iron oxide (MPIO) labeled MSCs and simultaneously to assess the effects on cardiac function after injection into the infarcted rat heart and the possible underlying mechanisms as well.

## 2. Materials and Methods

### 2.1. Animals

All animal studies were approved by the Institutional Animal Care and Use Committee at our hospital and all experiments were performed in accordance with the “Guide for the Care and Use of Laboratory Animals” published by the US National Institutes of Health (publication number 85-23, revised 1996).

### 2.2. MSCs Preparation and Labeling

Rat male MSCs were isolated and cultured using the methods as previously described [18]. A previous study has analyzed and tested the surface antigen profiles and potentials for multilineage differentiation [19]. In brief, we isolated MSCs from femurs and tibias of male Sprague Dawley rats (4 weeks, 60–80 g). The cells were subsequently seeded in Iscove's modified Dulbecco's medium (IMDM) culture medium (Gibco) with L-glutamine mixed with 10% fetal bovine serum (FBS, Gibco) on flasks. We replaced culture medium after 2 days and changed it twice a week afterward. 0.25% trypsin (EDTA, Sigma) was used to dissociate cells once they covered the 95% of the flask and then were replated to expand cells for successive passages. In terms of cell labeling, MSCs were incubated with fluorescent micron-sized particles of iron oxide (MPIO, 10 *μ*L of 1% stock solution per mL medium, Bangs Laboratories Inc.) in a humidified 5% CO_2_ incubator at 37°C for 24 h and followed by washing three times in phosphate-buffered saline (PBS) to rinse off spare MPIO particles. The label efficiency of MPIO was assessed by Prussian blue staining as well as microscopic examination. Before transplantation, cells were stained with CM-DiI (Invitrogen) in PBS for 30 minutes [20, 21]. The viability of the dual-labeled cells was assessed by trypan-blue exclusion before transfer. The transplanted MSCs were passage 3~4.

### 2.3. Myocardial Infarction and Cell Transfer

MI model was established using the method as previously described [18]. Briefly, SD female rats (200–250 g, *n* = 113) were anesthetized with intraperitoneal injection of chloral hydrate. Intubation and mechanical ventilation were subsequently performed using a small animal ventilation apparatus (model 683; Harvard Apparatus). With the heart exposed by a 2 cm lateral thoracotomy, the left coronary artery was permanently ligated a few millimeters below its origin with a 6-0 Prolene stitch. MI model was considered successfully established when myocardial blanching was visualized within the downstream myocardium. The thorax closure was done with three layers of sutures. 7 days after MI, the chest was reopened and rats were randomized to receive 1~2 sites direct injection of 2 × 10^6^/50 *μ*L dual-labeled MSCs (MSCs group) or 50 *μ*L PBS (control group) into the peri-infarct region.

### 2.4. MRI Protocol

Serial MRI was performed with a 7T horizontal-bore animal scanner (Varian NMR systems imaging with VnmrJ 2.1B software) at 24 h before cell transfer (baseline), 3 days, 2 and 4 weeks after cell transfer, respectively. Animals were anesthetized with isoflurane (3% for induction and 1.5–2% for maintenance) in oxygen. During image acquisition, small animal ECG electrodes (SA Instruments) were attached to the forelimbs of rats and a respiration-detection cushion was placed under the thorax in order to monitor and trigger ECG and respiration signals. For detecting the susceptibility artifacts (hypointensities) generated by the MPIO-labeled cells, T2^⁎^-weighted gradient echo sequence was acquired using the following parameters: FOV 40 × 40 mm, matrix 196 × 196, TE 2.83 ms, TR 56.32 ms, slice thickness 1.0 mm, and flip angle 20°. For assessing cardiac function, a stack of contiguous short-axis cine-MRI images were acquired to cover the entire left ventricle using the following parameters: FOV 40 × 40 mm, matrix 196 × 196, TE 1.8 ms, TR 10 ms, slice thickness 1.0 mm, 20° pulse, 4 averages, 10 cardiac phases, and 12 frames/cardiac cycle.

### 2.5. MRI Analysis

The left ventricular (LV) end-diastolic (EDV) and end-systolic (ESV) volumes were measured using ImageJ (NIH, Bethesda, MD). Stroke volume [8] was calculated as EDV minus ESV. LV ejection fraction (LVEF) was calculated as the SV divided by the EDV. The relative infarct size was calculated from the average of the endocardial and epicardial circumferential lengths of the thinned area of all slices, measured at diastolic stage and expressed as a percentage of the total myocardial surface. The signal intensity (SI) and hypointensive area were measured on the slice with maximum hypointensities area on T2^⁎^-weighted images using ImageJ (NIH, Bethesda, MD). A circular ROI of 11 pixels (0.70 mm^2^) was selected within hypointensive area and normal myocardium, respectively [20]. The signal contrast ratio was calculated as signal  contrast  ratio = (SI_normal  myocardium_ − SI_hypointensive  _area)/SI_normal  myocardium_ × 100%. Relative change (%) was calculated as [(follow-up parameter − baseline parameter)/baseline parameter] × 100.

### 2.6. Histology and Immunohistochemistry

At each time point after MRI scans, 3 animals from each group were sacrificed by overdose of pentobarbitone. Hearts were sectioned into 3 to 4 transverse slices and fixed with 4% buffered formalin, embedded in paraffin, and then sectioned with a microtome (5-*μ*m thick). Prussian blue staining was conducted to detect iron particles. To further identify the cells that had taken up the iron particles, antibody staining for the macrophage marker CD68 (Sigma) was performed in consecutive sections. Masson's-Trichrome staining was performed to assess myocardial fibrosis, and the fibrosis score was calculated as collagen fiber area/total view area × 100%. Apoptosis was assessed by the terminal deoxynucleotidyl transferase-mediated dUTP nick end-labeling (TUNEL) assay kit (Roche, Indiana, USA), and the apoptosis score was calculated as the number of TUNEL-positive cardiomyocytes/the total number of cardiomyocytes × 100%. Angiogenesis was assessed by counting the number of vessels immunostained for the endothelial cell marker CD31 (rabbit polyclonal, Abcam, UK). Random fields (*n* = 20) around each peri-infarct area were selected and numbers of vessels were counted.

### 2.7. Transmission Electron Microscopy

The heart tissues were cut into approximately 1 mm cubes and fixed with 2.5% glutaraldehyde in 0.1 M phosphate buffer at 4°C for 2 h. After washing with sodium cacodylate buffer, samples were postfixed with 1% osmium tetroxide at 4°C for 2 h. Samples were then dehydrated by a serial gradient ethanol and then embedded in Epon-812 (Electron Microscopy Sciences). Ultrathin sections were cut using an ultramicrotome (Leica, Leica EM UC7) on uncoated copper grids and stained with 0.2% lead citrate/1% uranyl acetate. Images were recorded under a transmission electron microscope (JEM1400; JEOL).

### 2.8. Real-Time PCR

For assessing the MSCs retention after transplantation, RT-PCR analysis for the rat Y-chromosome-specific SRY gene was performed from infarcted hearts of 3 female recipients treated with male MSCs at each time point. Total RNA was extracted by the Trizol reagent method (Invitrogen), and first strand cDNA was synthesized with SuperScript II (Invitrogen). The primer sequence for rat SRY gene were forward primer 5′-AGGGTTAAAGTGCCACAGAGGA-3′ and reverse primer 5′-GCTTTT CTGGTTCTTGGAGGAC-3′. The primers and probe for GAPDH gene were forward primer 5′-AACCTGCCAAGTATGATGACATCA-3′ and reverse primer 5′-TTCCACTGATATCCCAGCTGCT-3′. PCR products were electrophoresed through 1.5% agarose gels containing ethidium bromide. Quantitative real-time PCR involved the iCycler iQ system (Bio-Rad).

### 2.9. Statistical Analyses

Data were presented as means ± standard deviation. Differences between the two groups were made using unpaired Student's *t*-test; Statistical comparisons among different time points in each group were made using repeated measures ANOVA. All statistical analyses were conducted using SPSS 16.0 (SPSS, Chicago, IL). A value of *p* < 0.05 was considered significant.

## 3. Results

### 3.1. Establishment of Myocardial Infarction Model and Mortality

Myocardial infarction (MI) surgery was successfully performed on 113 rats. Of them, 20 died within 24 hours of the surgical operations (17% postoperative mortality) due to procedure-related complications. Absence of or minimal MI was revealed by baseline MRI (defined as LVEF > 60%) in 7 rats, which were further excluded in the study. Therefore, the remaining qualified 86 rats were randomized into two groups, MSCs engrafted and control group. During cell injection, a total of 16 rats died, 9 in the MSCs group and 7 in the control group. Thus, 70 rats were included in the final analysis (34 rats in the MSCs group, 36 rats in the control group).

### 3.2. MSCs Labeling and Morphology

**Figure 1:**
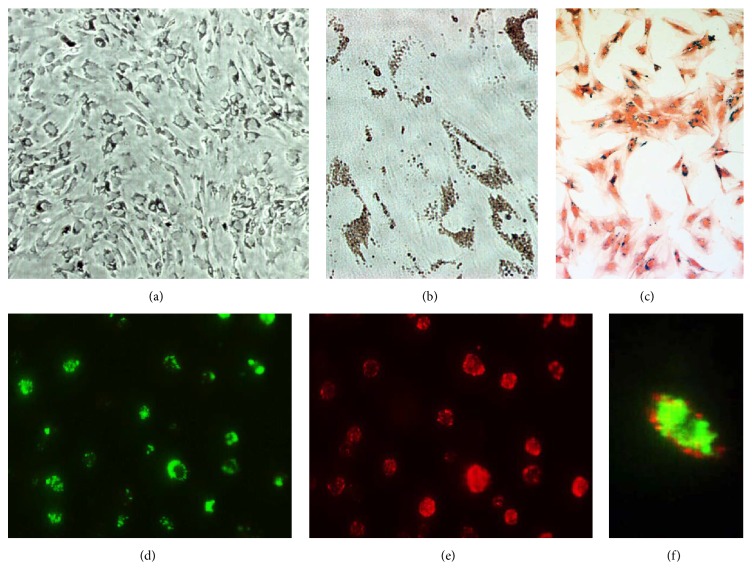
Dual-labeled mesenchymal stem cells (MSCs). (a) Phase contrast images showed almost 99% of the cells labeled with MPIO (original magnification ×100). (b) The iron particles gathered in the cytoplasm and perinuclear area (original magnification ×400). (c) Iron staining demonstrated high MPIO labeling efficiency (>98%) represented as numerous blue granules in the cytoplasm (original magnification ×100). (d)–(f) The green fluorescence of the MPIOs was clearly detected in the cytoplasm (d and f) and the red CM-DiI in the membrane of MSCs (e and f), original magnification ×400.

MSCs were efficiently labeled (>98%) in culture at a concentration of 10 *μ*L MPIO stock per mL of medium for 24 h, confirmed by phase contrast microscopy (Figures 1(a) and 1(b)) and iron staining (Figure 1(c)). Fluorescence microscopy demonstrated MSCs were efficiently dual-labeled with MPIO and CM-DiI. The dragon green fluorescence of the MPIOs was clearly detected in the cytoplasm and the red of CM-DiI in the membrane of cells (Figures 1(d)–1(f)). By trypan-blue exclusion assay, almost 99% of the cells remained viable before transplantation.

### 3.3. MRI Measurement of Cardiac Morphology and Function

**Figure 2:**
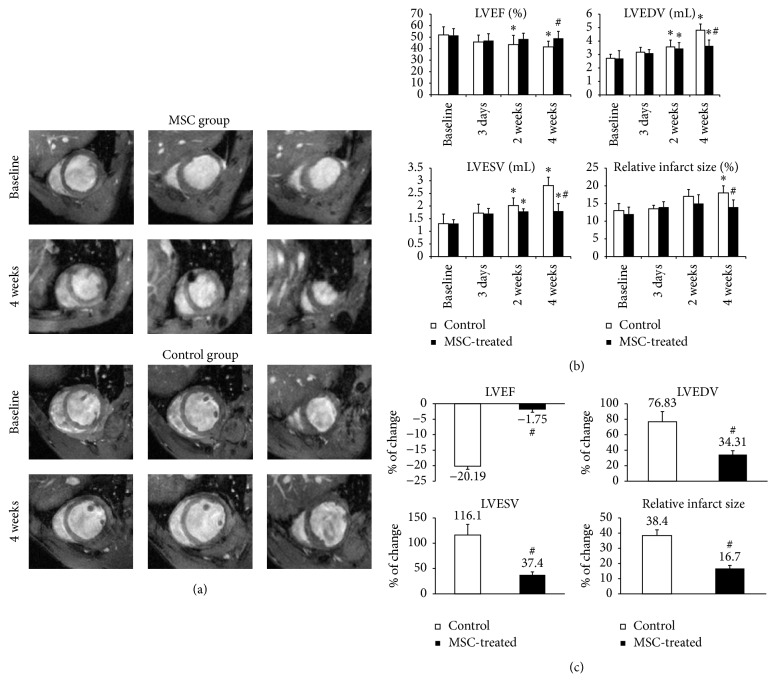
MRI assessment of cardiac morphology and function. (a) The typical course after myocardial infarction including progressive scar thinning, left ventricular dilatation, and functional deterioration was observed in both groups at baseline. However, serial cine-MRI studies between baseline and 4 weeks showed that MSCs injection resulted in moderated LV dilatation and dysfunction compared with controls (b–c). *N* = 9, 9,8, 8 for MSCs group and *N* = 10, 9,9, 8 for control group at each time point. ^*∗*^ *p* < 0.05 versus baseline; ^#^ *p* < 0.05 versus control.

Cine images were acquired to assess the cardiac morphology and function at baseline (24 h before transplantation), 3 days, and 2 and 4 weeks after cell injection. The typical course after myocardial infarction including progressive scar thinning, left ventricular dilatation and functional deterioration were observed in both groups at baseline (Figure 2(a)), and there were no significant differences between the two groups in terms of LVEF, EDV, and ESV. Although both groups showed pronounced LV dilatation, both EDV and ESV in MSCs group were significantly smaller than that of control group 4 weeks after transplantation (3.6 ± 0.4 mL versus 4.8 ± 0.4 mL, *p* < 0.05 and 1.8 ± 0.4 mL versus 2.8 ± 0.3 mL, *p* < 0.05, resp.). Meanwhile, the LVEF in control group was significantly lower than that of MSCs-treated hearts (41 ± 5% versus 49 ± 6%, *p* < 0.05). Relative infarct size was similar between the groups at the first 3 time points, which then increased significantly in control hearts but not in MSCs-treated hearts at 4 weeks (18 ± 2% versus 14 ± 2%, *p* < 0.05) (Figure 2(b)). Furthermore, the relative changes of LVEF, EDV, ESV, and infarct size in MSCs group between baseline and 4 weeks after cell delivery were significantly smaller than controls (Figure 2(c)).

### 3.4. MRI Tracking of the Injected MSCs

**Figure 3:**
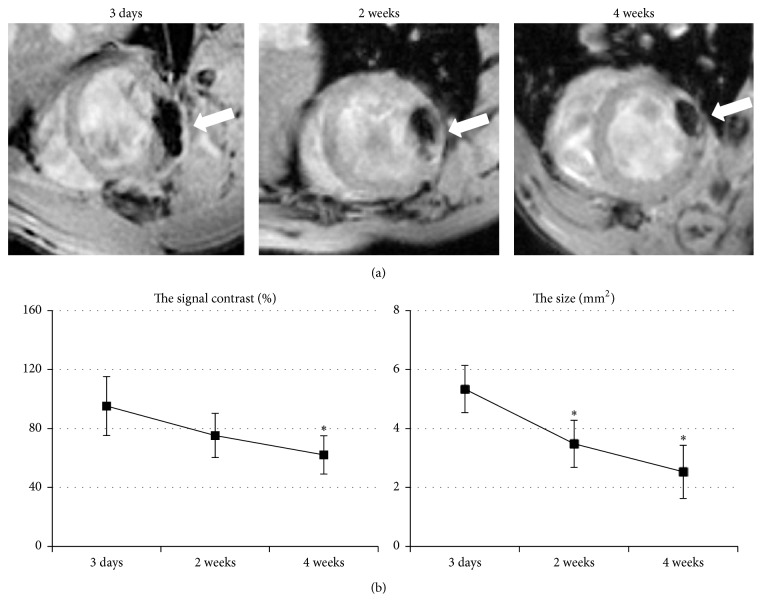
In vivo tracking of the MSCs after transplantation. (a) Persistent MR hypointensities caused by MPIOs were detected at all time points at the site of injection after transplantation (arrows). (b) As time progressed, the signal gradually weakened and the area decreased. *N* = 9,9, 8,8 at each time point ^*∗*^ *p* < 0.05 versus 3 days.

T2^⁎^-weighted images were acquired to in vivo tracking MPIO-labeled cells at 3 days and 2 and 4 weeks after injection into the peri-infarct region of LV anterior wall, respectively. Persistent hypointensities (“black spots”) generated by MPIO particles were detected in all animals that received MSCs transplantation. 3 days later, large well-defined hypointensities which extended beyond the whole LV wall could be visualized at the site of injection, while the virtual area of MPIO-labeled cells was smaller, which was called blooming effect. However, as time progressed, the signal of “black spot” gradually weakened and the area decreased (Figure 3(a)). There was a significant decrease both in signal contrast ratio (%) and in the low signal area (mm^2^) at 4 weeks (95.27 ± 20.10 versus 62.14 ± 13.58 and 5.34 ± 0.80 versus 2.53 ± 0.93, *p* < 0.05, resp.) than those at 3 days after transfer; no statistic differences were observed between 3 days and 2 weeks (Figure 3(b)). For control hearts, no “black spot” was detected on T2^⁎^ weighted images.

### 3.5. Distribution and Retention of Injected MSCs in Hearts

**Figure 4:**
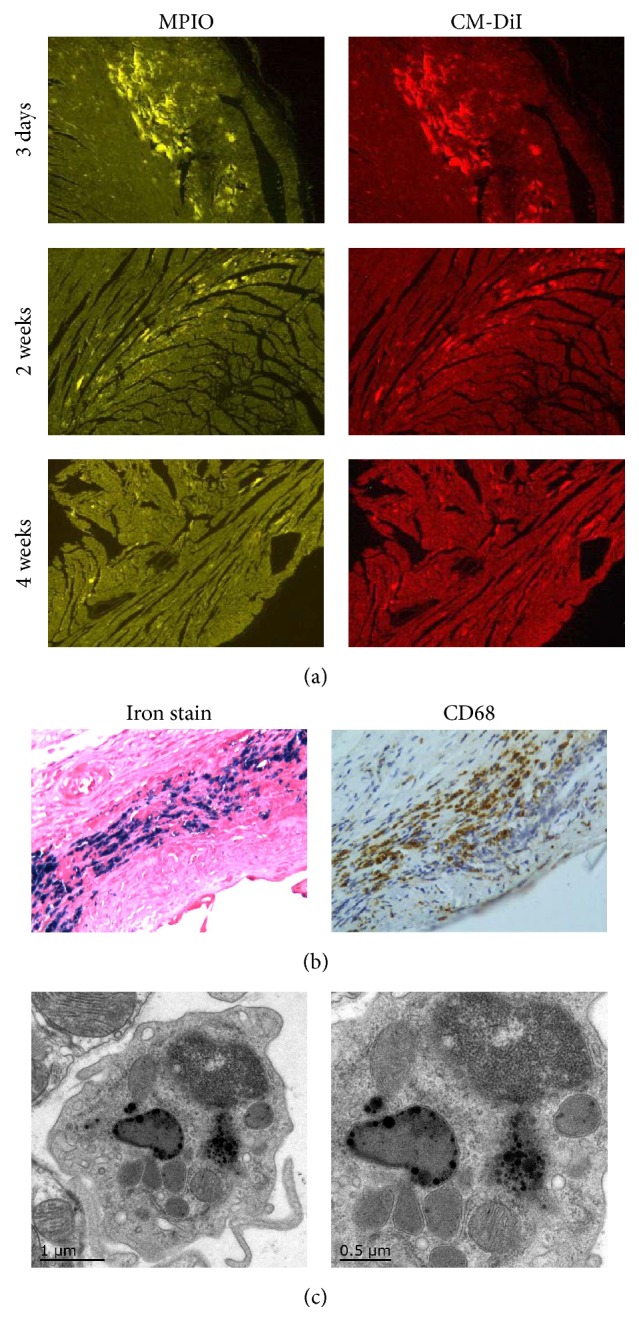
MSCs retention in recipient hearts. (a) The number of dual-labeled MSCs decreased over time. At 4 weeks, almost no positive cells were detected (original magnification ×50). (b) Sections stained for iron and CD68 at 4 weeks showed that most of the iron-positive cells (blue cytoplasm) were CD68-positive cardiac macrophages (brown cytoplasm) (original magnification ×200). (c) Representative electron micrographs demonstrated MPIOs engulfment inside a macrophage.

The detection of dual-labeled MSCs was confirmed by colocalization of two markers, green fluorescent MPIOs and red CM-DiI at the site of MSCs injection, which demonstrated successful cell transplantation. However, the number of positive cells significantly reduced over time, and almost no positive dual-labeled cells were found in MSCs group at 4 weeks after cell delivery (Figure 4(a)). Double staining for iron and CD68 (a marker of resident macrophage) at 4 weeks revealed that most iron-positive cells were also CD68 positive (Figure 4(b)). This was further confirmed by transmission electron microscopy, which demonstrated iron particles inside the macrophages (Figure 4(c)). Consistently, Real-time PCR analysis for the rat Y-chromosome-specific SRY gene demonstrated the retention of survival MSCs reduced significantly after injection: 3 days after the injection of 2 × 10^6^ MSCs (*n* = 3), 11.5% of the initially engrafted cells were detected. After 2 weeks, this number dropped to 1.2% (*n* = 3) and further declined to ~0.1% at 4 weeks (*n* = 3).

### 3.6. Cardiac Fibrosis, Apoptosis, and Capillary Density in Peri-Infarcted Region

**Figure 5:**
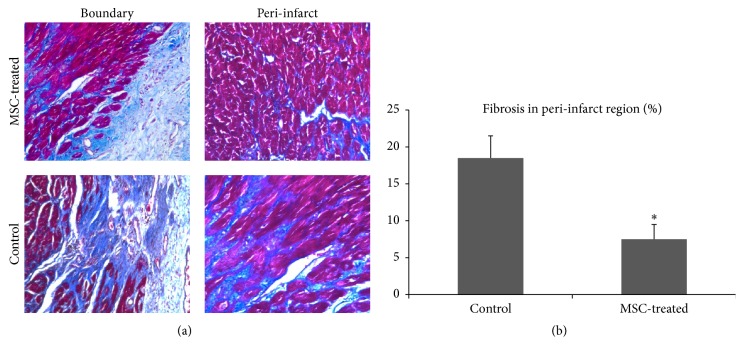
Myocardial fibrosis in peri-infarct area 4 weeks after transplantation. (a) Masson staining showed that, in control hearts, the border of myocardial infarction was more irregular and the fibrosis was more pronounced (original magnification ×200). (b) Quantification of fibrosis. *N* = 3 for each group, ^*∗*^ *p* < 0.05 versus control.

**Figure 6:**
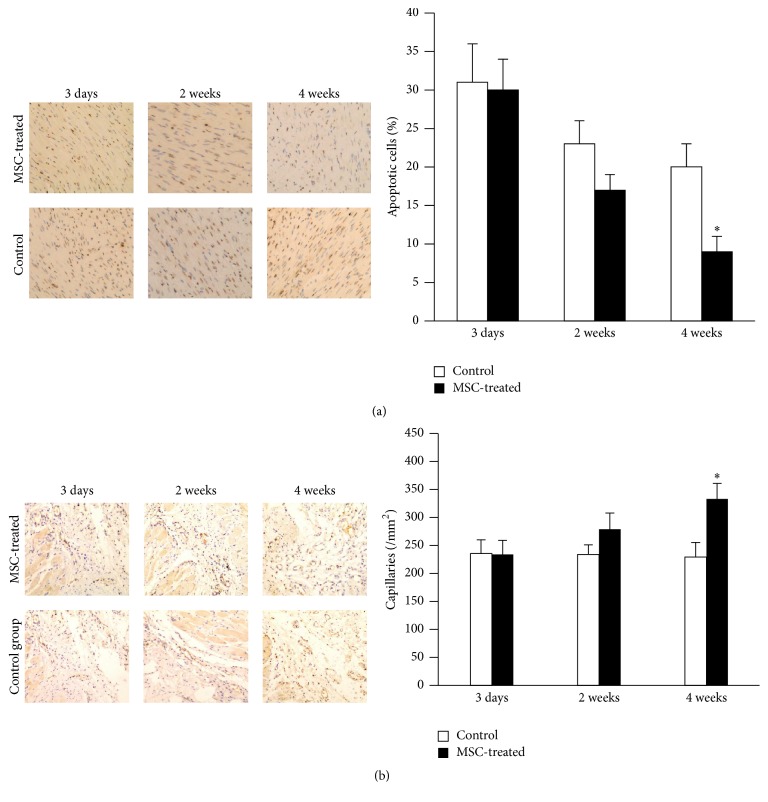
Cardiomyocytes apoptosis and capillary density in peri-infarct area after transplantation. (a) TUNEL staining and apoptosis. (b) CD31 staining and capillary density (original magnification ×200). *N* = 3 for each group and ^*∗*^ *p* < 0.05 versus control.

The degree of fibrosis determined by Masson staining was much severer in the control group, and the fibrosis score was significantly higher than that in MSCs group (18.5 ± 3.1% versus 7.5 ± 2.2%, *p* < 0.05) (Figure 5). TUNEL staining demonstrated high level of apoptosis in both groups after MI. But MSCs injection significantly reduced apoptotic cell death at 4 weeks after transplantation (9.4 ± 2.1% versus 20.3 ± 5.2%, *p* < 0.05) (Figure 6(a)). CD31 staining showed that no significant difference regarding the number of capillaries was observed between the two groups until 4 weeks. Compared with the control group (255.3 ± 29.1/mm^2^), the number of capillaries was significantly increased by 30% (*p* < 0.05) in the MSC-treated hearts (370.6 ± 31.5/mm^2^) at 4 weeks (Figure 6(b)).

## 4. Discussion

The main findings of current study are that iron particles are not a reliable marker for in vivo tracking the long-term fate of MSCs engraftment. However, despite poor cell retention, MSCs moderate left ventricular remodeling after MI due to the enhanced angiogenesis, inhibition of cardiomyocytes apoptosis, and fibrosis.

MSCs transplantation is a promising method to treat myocardial infarction and heart failure due to their proliferation and differentiation potentials. Understanding the fate of grafted stem cells is critical not only in the development of effective stem cell therapies but also in understanding the underlying mechanisms of therapeutic benefits. Therefore, a sensitive, noninvasive imaging technology is desperately needed for in vivo tracking the transplanted stem cells regarding temporal cell location, distribution, viability, and functional status. MRI is currently considered a standard tool to assess the cardiac structure and function due to its versatility, accuracy, and reproducibility and has been most frequently applied in the clinical trials [6–8, 22].

Several researchers have demonstrated that MSCs transplantation can improve cardiac function and reduce the degree of scar tissue [23–25], supported by our results. But there were other studies reporting a negative or no discernable functional benefits between MSCs-treated and control hearts [26–28]. Reasons for the inconsistencies include all aspects of cell therapy such as different levels of cardiac impairment, species and animal age, variations in the time, and methods of cell delivery and the number of cells injected. In this study, we injected MSCs at 7 days after MI instead of 2 weeks applied in our previous study [17], which resulted in a significant functional improvement by MSCs injection. The benefits were expected according to a research by Hu et al. [29], focusing on the optimal timing of cell transplantation after MI. They found that the functional improvement was significantly pronounced with injection at 1 week after MI compared to any other time points including immediately and 2 weeks after MI, because at that moment the inflammatory responses were much weaker and the scar tissue had not yet formed. A further explanation for the discrepancy between studies is that different imaging tools have been used to assess cardiac function. Echocardiography has been regularly used to measure cardiac function in rodent hearts due to their convenience. However, its accuracy and reproducibility are controversial [16], and the measurements are less accurate than cine-MRI when applied to asymmetric, infarcted rat hearts. However, MRI is not commonly used to study rodent heart function due to their small size and quite high heart rate (>300 beats/min). The 7T cine-MRI method used in this study yielded excellent contrast between myocardium and blood and resulted in high-definition, volumetric images from which ESV and EDV could be accurately measured [16, 30, 31].

Studies have reported that MRI can in vivo track the fate of grafted stem cells labeled with MPIO, and the MPIOs do not affect cell viability, proliferation, differentiation, or therapeutic effects [32–34]. However, several studies have challenged the reliability of iron-particle tracking the transplanted stem cells [13, 18], because iron particles may be engulfed by macrophages after stem cells died and decomposed, which may interfere with MRI to distinguish the survival stem cells from the dead ones. In line with our previous study [17], we also demonstrated that the number of survival MSCs decreased progressively over time after successful cell injection, and iron particles distribution was consistent with the macrophages. These findings indicated that MRI could not identify whether the MPIOs arose from the survival MSCs or macrophages. Therefore, we suggested that iron particles are not a reliable marker to monitor the transplanted stem cells viability and retention but localize the injection site in vivo.

Of note, although the cell retention was quite poor, MSCs maintained their protective effect against progressive LV remodeling and dysfunction after myocardial infarction. The underlying mechanisms of this functional improvement remain unclear; previous reports suggested that the therapeutic effects of transplanted cells on LV remodeling and function might be independent of cell retention or transdifferentiation [24, 35]. One recent study showed that intramyocardial transplantation of MSCs after acute MI could improve cardiomyocytes' glucose metabolism and cardiac function [36]. According to their results, the potential mechanism of this phenomenon might be the simultaneous activation of mTOR signal transduction pathways through paracrine, thus promoting myocardial glucose metabolism and ATP production, which leads to improvement of cardiac function. Other study found the enhancement of angiogenesis via paracrine effect with increased expression of vascular endothelial growth factor (VEGF) resulted in the improved myocardial function [37]. Similarly, our results also revealed MSCs injection significantly increased angiogenesis and reduced myocardial apoptosis and collagen deposition in the peri-infarct region. We speculated that secretion of certain cytokines might contribute to the paracrine effects, which potentially plays a critical role in the therapeutic effects on the infarcted hearts rather than transdifferentiation in this case [38, 39].

This study has several limitations. First, the relatively short-term observation prevented definite conclusion of long-term effects of cell therapy. Second, myocardial perfusion imaging was not performed to further confirm the blood flow restoration due to angiogenesis in the MSCs group. Finally, additional laboratory analyses were required to further identify the underlying mechanisms.

In conclusion, iron particles are not a reliable marker for in vivo tracking the long-term fate of MSCs engraftment. Despite poor cell retention, MSCs moderate left ventricular remodeling after MI due to the enhanced angiogenesis, inhibition of cardiomyocytes apoptosis, and fibrosis.

## References

[1] Pittenger M. F., Martin B. J. (2004). Mesenchymal stem cells and their potential as cardiac therapeutics. *Circulation Research*.

[2] Povsic T. J., O'Connor C. M. (2010). Cell therapy for heart failure: the need for a new therapeutic strategy. *Expert Review of Cardiovascular Therapy*.

[3] Stamm C., Westphal B., Kleine H.-D. (2003). Autologous bone-marrow stem-cell transplantation for myocardial regeneration. *The Lancet*.

[4] Menasché P., Hagège A. A., Vilquin J.-T. (2003). Autologous skeletal myoblast transplantation for severe postinfarction left ventricular dysfunction. *Journal of the American College of Cardiology*.

[5] Fernández-Avilés F., San Román J. A., García-Frade J. (2004). Experimental and clinical regenerative capability of human bone marrow cells after myocardial infarction. *Circulation Research*.

[6] Wollert K. C., Meyer G. P., Lotz J. (2004). Intracoronary autologous bone-marrow cell transfer after myocardial infarction: the BOOST randomised controlled clinical trial. *The Lancet*.

[7] Janssens S., Dubois C., Bogaert J. (2006). Autologous bone marrow-derived stem-cell transfer in patients with ST-segment elevation myocardial infarction: double-blind, randomised controlled trial. *The Lancet*.

[8] Lunde K., Solheim S., Aakhus S. (2006). Intracoronary injection of mononuclear bone marrow cells in acute myocardial infarction. *The New England Journal of Medicine*.

[9] Uemura R., Xu M., Ahmad N., Ashraf M. (2006). Bone marrow stem cells prevent left ventricular remodeling of ischemic heart through paracrine signaling. *Circulation Research*.

[10] Olivares E. L., Costa-e-Sousa R. H., Werneck-de-Castro J. P. S. (2007). Cellular cardiomyoplasty in large myocardial infarction: can the beneficial effect be enhanced by ACE-inhibitor therapy?. *European Journal of Heart Failure*.

[11] Peng C., Yang K., Xiang P. (2013). Effect of transplantation with autologous bone marrow stem cells on acute myocardial infarction. *International Journal of Cardiology*.

[12] Nagaya N., Fujii T., Iwase T. (2004). Intravenous administration of mesenchymal stem cells improves cardiac function in rats with acute myocardial infarction through angiogenesis and myogenesis. *American Journal of Physiology—Heart and Circulatory Physiology*.

[13] Amsalem Y., Mardor Y., Feinberg M. S. (2007). Iron-oxide labeling and outcome of transplanted mesenchymal stem cells in the infarcted myocardium. *Circulation*.

[14] Buck T., Hunold P., Wentz K. U., Tkalec W., Joachim Nesser H., Erbel R. (1997). Tomographic three-dimensional echocardiographic determination of chamber size and systolic function in patients with left ventricular aneurysm: comparison to magnetic resonance imaging, cineventriculography, and two- dimensional echocardiography. *Circulation*.

[15] Grothues F., Smith G. C., Moon J. C. C. (2002). Comparison of interstudy reproducibility of cardiovascular magnetic resonance with two-dimensional echocardiography in normal subjects and in patients with heart failure or left ventricular hypertrophy. *The American Journal of Cardiology*.

[16] Stuckey D. J., Carr C. A., Tyler D. J., Clarke K. (2008). Cine-MRI versus two-dimensional echocardiography to measure in vivo left ventricular function in rat heart. *NMR in Biomedicine*.

[17] Ma N., Cheng H., Lu M. (2015). Magnetic resonance imaging with superparamagnetic iron oxide fails to track the long-term fate of mesenchymal stem cells transplanted into heart. *Scientific Reports*.

[18] Winter E. M., Hogers B., van der Graaf L. M., Gittenberger-de Groot A. C., Poelmann R. E., van der Weerd L. (2010). Cell tracking using iron oxide fails to distinguish dead from living transplanted cells in the infarcted heart. *Magnetic Resonance in Medicine*.

[19] Wang W., Jiang Q., Zhang H. (2011). Intravenous administration of bone marrow mesenchymal stromal cells is safe for the lung in a chronic myocardial infarction model. *Regenerative Medicine*.

[20] Hill J. M., Dick A. J., Raman V. K. (2003). Serial cardiac magnetic resonance imaging of injected mesenchymal stem cells. *Circulation*.

[21] Yang Y., Schumacher A., Yang Y. (2011). Monitoring bone marrow-originated mesenchymal stem cell traffic to myocardial infarction sites using magnetic resonance imaging. *Magnetic Resonance in Medicine*.

[22] Meyer G. P., Wollert K. C., Lotz J. (2006). Intracoronary bone marrow cell transfer after myocardial infarction: eighteen months' follow-up data from the randomized, controlled BOOST (Bone marrow transfer to enhance ST-elevation infarct regeneration) trial. *Circulation*.

[23] Zhang S., Ge J., Sun A. (2006). Comparison of various kinds of bone marrow stem cells for the repair of infarcted myocardium: single clonally purified non-hematopoietic mesenchymal stem cells serve as a superior source. *Journal of Cellular Biochemistry*.

[24] Balsam L. B., Wagers A. J., Christensen J. L., Kofidis T., Weissmann I. L., Robbins R. C. (2004). Haematopoietic stem cells adopt mature haematopoietic fates in ischaemic myocardium. *Nature*.

[25] Kocher A. A., Schuster M. D., Szabolcs M. J. (2001). Neovascularization of ischemic myocardium by human bone-marrow-derived angioblasts prevents cardiomyocyte apoptosis, reduces remodeling and improves cardiac function. *Nature Medicine*.

[26] Dai W., Hale S. L., Martin B. J. (2005). Allogeneic mesenchymal stem cell transplantation in postinfarcted rat myocardium: short- and long-term effects. *Circulation*.

[27] Deten A., Volz H. C., Clamors S. (2005). Hematopoietic stem cells do not repair the infarcted mouse heart. *Cardiovascular Research*.

[28] Mangi A. A., Noiseux N., Kong D. (2003). Mesenchymal stem cells modified with Akt prevent remodeling and restore performance of infarcted hearts. *Nature Medicine*.

[29] Hu X., Wang J., Chen J. (2007). Optimal temporal delivery of bone marrow mesenchymal stem cells in rats with myocardial infarction. *European Journal of Cardio-Thoracic Surgery*.

[30] Beeres S. L. M. A., Bengel F. M., Bartunek J. (2007). Role of imaging in cardiac stem cell therapy. *Journal of the American College of Cardiology*.

[31] Fuster V., Sanz J., Viles-Gonzalez J. F., Rajagopalan S. (2006). The utility of magnetic resonance imaging in cardiac tissue regeneration trials. *Nature Clinical Practice Cardiovascular Medicine*.

[32] Carr C. A., Stuckey D. J., Tatton L. (2008). Bone marrow-derived stromal cells home to and remain in the infarcted rat heart but fail to improve function: an in vivo cine-MRI study. *American Journal of Physiology—Heart and Circulatory Physiology*.

[33] Drey F., Choi Y.-H., Neef K. (2013). Noninvasive in vivo tracking of mesenchymal stem cells and evaluation of cell therapeutic effects in a murine model using a clinical 3.0 T MRI. *Cell Transplantation*.

[34] Kraitchman D. L., Heldman A. W., Atalar E. (2003). In vivo magnetic resonance imaging of mesenchymal stem cells in myocardial infarction. *Circulation*.

[35] Agbulut O., Vandervelde S., Al Attar N. (2004). Comparison of human skeletal myoblasts and bone marrow-derived CD133^+^ progenitors for the repair of infarcted myocardium. *Journal of the American College of Cardiology*.

[36] Cai M., Shen R., Song L. (2016). Bone Marrow Mesenchymal Stem Cells (BM-MSCs) improve heart function in swine myocardial infarction model through paracrine effects. *Scientific Reports*.

[37] Tse H.-F., Siu C.-W., Zhu S.-G. (2007). Paracrine effects of direct intramyocardial implantation of bone marrow derived cells to enhance neovascularization in chronic ischaemic myocardium. *European Journal of Heart Failure*.

[38] Kinnaird T., Burnett E. S., Shou M. S. (2004). Local delivery of marrow-derived stromal cells augments collateral perfusion through paracrine mechanisms. *Circulation*.

[39] Gnecchi M., He H., Liang O. D. (2005). Paracrine action accounts for marked protection of ischemic heart by Akt-modified mesenchymal stem cells. *Nature Medicine*.

